# State of Medicine in Spain

**Published:** 1846-02

**Authors:** 


*State of Medicine in Spain.* 1845.—Unfortunate the wight who falls ill in Spain, as, whatever his original complaint, it is too often followed by secondary and worse symptoms, the native doctor. The faculty at Madrid are little in advance of their provincial colleagues, —nay, often they are more destructive, since, being practitioners *en la Corte*, the heaven on earth, they are in proportion superior to the medical men of the rest of the world, of whom of course they can learn nothing. They are, however, at least a century behind the practitioners of England. Their notions and practice are classical, oriental, and antiquated, and their acquaintance with modern works, inventions, and operations, very limited. Their text-books and authorities are Galen, Celsus, Hippocrates, and Boerhave; the names of Hunter, Harvey, and Astley Cooper, are scarcely more known among their M. D.s than the last discoveries of Herschel; the light of such distant planets has not had time to arrive.

Meanwhile, as in courts of justice and other matters in Spain, all sounds admirably on *paper*—the forms, regulations, and system are perfect in theory. Colleges of physicians and surgeons superintend the science; the professors are members of learned societies; lectures are delivered, examinations are conducted, and certificates, duly signed and sealed, are given. The young *Galenista* is furnished with a license to kill. What is wanting from beginning to end, to practitioner and patient, is *life*. The salaries of teachers are ill paid, and the pupils are tampered with and their studies thought dangerous, not to private but the public weal; thus Ferdinand VII., on the news of the three glorious days of Paris, shut up the medical schools, opening, it is true, by way of compensation, a university for killing bulls *secundem artem*. The medical men know, nevertheless, every aphorism of the ancients by rote, and *discourse* as eloquently and plausibly on any case as do their ministers in the Cortes. Both write capital *documentos*, theories and opinions, extemporaneously. Their splendid language supplies words which seem to have cost thought. What is wanting is *practice*, and that clinical and best of education where the case is brought before the student with the corollary of skilful treatment.

As in their modern art and literature, there is little originality in Spanish medicine. It is chiefly a veneering of other men's ideas, or an adaptation of ancient and Moorish science. Most of their technical terms, *jalea*, *elixir*, *jarave*, *rob*, *sorbete*, *juleps*, *&c.*, are purely Arabic, and indicate the sources from whence the knowledge was obtained; and whenever they depart from the daring ways of their ancestors, it is to adopt a timid French system. The few additions to their medical libraries are translations from their neighbors, just as the scanty materia medica in their apothecaries' shops is rendered more ineffective by quack nostrums from Paris. In spite of these lamentable deficiencies, the self-esteem of the medical men exceeds, if possible, that of the military; both have killed their "ten thousands." They hold themselves to be the first *sabreurs*, physicians, and surgeons on earth, and best qualified to wield the shears of the

Parcæ. It would be a waste of time to try to dispel this fatal delusion; the well-intentioned monitor would simply be set down as malevolent, envious, and an ass; for they think their ignorance the perfection of human skill. No foreigner can ever hope to succeed among them, nor can any native who may have studied abroad easily introduce a better system. All his brethren would make common cause against him as an innovator. He would be summoned to no consultations, the most lucrative branch of practice, while the confessors would poison the ears of the women (who govern the men,) with cautions against the danger to their souls of having their bodies cured by a Jew, a heretic, or a foreigner, for the terms are almost convertible.

Dissection is now repulsive to their oriental prejudices; the pupils learn rather by plates, diagrams, models, preparations, and skeletons, than from anatomical experiments on a subject; their practice necessarily is limited. In difficult cases of compound fracture, gunshot wounds, the doctors give the patient up almost at once, although they continue to meet and take fees until death relieves him of his complicated sufferings. In chronic cases and slighter fractures they are less dangerous; for as their pottering remedies do neither good nor harm, the struggle for life and death is left to nature, who sometimes works the cure. In acute diseases and inflammations they seldom succeed; for however fond of the lancet, they only nibble with the case, and are scared at the bold decided practice of Englishmen, whereat they shrug up shoulders, invoke saints, and descant learnedly on the impossibility of treating complaints under the bright sun and warm air of catholic Spain, after the formulæ of cold, damp, and foggy, heretical England.

Most Spaniards who can afford it, have their family doctor, or *Medico de Cabecera*, and their confessor. This pair take care of the bodies and souls of the whole house, bring them gossip, share their *puchero*, purse, and tobacco. They rule the husband through the women and the nursery; nor do they allow their exclusive privileges to be infringed on. Etiquette is the life of a Spaniard, and often his death. Every one knows that Philip III. was killed, rather than violate a form. He was seated too near the fire, and although burning, of course as king of Spain the impropriety of moving himself never entered his head; and when he requested one of his attendants to do so, none, in the absence of the proper officer whose duty it was to superintend the royal chair, ventured to take that improper liberty. In case of sudden emergencies among her Catholic Majesty's subjects, unless the family doctor be present, any other one, even if called in, generally declines acting until the regular Esculapius arrives. An English medical friend of ours saved a Spaniard's life by chancing to arrive when the patient, in an apoplectic fit, was foaming at the mouth and wrestling with death; all this time a strange doctor was sitting quietly in the next room smoking his cigar at the *brasero* with the women of the family. Our friend instantly took thirty ounces from the sufferer's arm, not one of the Spanish party even moving from their seats, hunc sic servavit Apollo!

The Spanish medical men pull together—a rare exception in Spain—and play into each other's hands. The family doctor, whenever appearances will in anywise justify him, becomes alarmed, and requires a consultation, a *Junta*. What any Spanish Junta is, need not be explained; and these are like the rest, they either do nothing or what they do, is done badly. At these meetings from three to seven *Medicus de apelacion*, consulting physicians, attend, or more, according to the patient's purse; each goes to the sick man, feels his pulse, asks him some questions, and then retires to the next room to consult, generally allowing the invalid the benefit of hearing what passes. The *Protomedico*, or senior, takes the chair; and while all are lighting their cigars, the family doctor opens the case, by stating the birth, parentage, and history of the patient, his constitution, the complaint, and the medicines hitherto prescribed. The senior next rises, and gives his opinion, often speaking for half an hour; the others follow in their rotation, and then the *Protomedico*, like a judge, sums up, going over each opinion with comments; the usual termination is either to confirm the previous treatment, or order some insignificant *tisana;* the only certain thing is to appoint another consultation for the next day, for which the fees are heavy, each taking from three to five dollars. The consultation often lasts many hours, and is a chronic complaint. It occurred to our same medical friend to accidentally call on a person who had an inflammation in the cornea of the eye; on questioning he found that many consultations had been previously held, at which no determination was come to until at the last, when sea-bathing was prescribed, with a course of asses' milk and Chiclana snake-broth; our heretical friend, who lacked the true *Fe*, just touched the diseased part with caustic. When this application was reported at the next *Junta*, the *Medicos* all crossed themselves with horror and amazement, which was increased when the patient recovered in a week.

The trade of a druggist is anything but free; none may open a *Botica* without a strict examination and license; of course this is to be had for money. None may sell any potent medicine, except according to the prescription of some *local* medical man; every thing is a monopoly. The commonest drugs are often either wanting or grossly adulterated, but, as in their arsenals and larders, no dispenser will admit such destitution; *hay de todo*, swears he, and gallantly makes up the prescription simply by substituting other ingredients; and as the correct ones nine times out of ten are harmless, no great injury is sustained; if, by chance, the patient dies, the doctor and the disease bear the blame. Perhaps the old Iberian custom was the safest; the sick were exposed outside their doors, and the advice of casual passengers was asked (Strabo, iii., 324,) whose prescriptions were quite as likely to answer as images, relics, *bouillon aux vipres*, or milk of almonds or asses.

The poor and more numerous class, especially in the rural districts, seldom use physic—oh fortunati nimium! Like their mules they are rarely ill; they only take to their beds to die. If they do consult any one, it is the barber, the quack, or *curandero;* for there is generally in Spain some charlatan wherever sword, rosary, pen, or lancet is to be wielded. The nostrums, charms, relics, incantations, &c., to which recourse is had, when not mediæval, are pagan. For the spiritual pharmacopœia see Sa, Engracia's lamp oil and our remarks (Zaragoza.) The patients cannot always be expected to recover even then, since "*Para todo hay remedio, sino para la muerte.*"—"There is a remedy for everything except death." The transition from surgeons to barbers is easy in Spain; nay, shaving in this land, where whiskers were the type of valour and knighthood, long took the precedence over surgery; and even now, the shops of the Figaros are far more interesting than the hospitals. Here most ludicrous experiments are tried on the teeth and veins of the brave vulgar. The *Tienda de Barbero* is distinguished by a Mambrino's helmet basin, by phlebotomical symbols, and generally a rude painting of bleeding at the foot; huge grinders are hung up, which in a church would be exhibited as relics of St. Christopher; inside is a guitar and prints of bull-fights, while Figaro, the centre of all, is the personification of bustle and gossip. Few Spaniards can shave themselves; it is too mechanical, even supposing their cut,lerscould make a razor. Like orientals, they prefer a "razor that is hired." These Figaros shave well, but not silently, the request of the Andaluz Adrian; garrulous by nature and trade, they have their own way in talk; for when a man is in their operating chair, with his jaws lathered, his nose between a finger and thumb, and a sharp blade at his throat, there is not much conversational fair play or reciprocity.—*Ford's Spain.—Med. Gazette.*

